# Functional validation of human-specific PowerPlex^®^ 21 System (Promega, USA) in chimpanzee (*Pan troglodytes*)

**DOI:** 10.1186/s13104-018-3803-x

**Published:** 2018-10-03

**Authors:** Mukesh Thakur, Kailash Chandra, Vivek Sahajpal, Asis Samanta, Arun Sharma, Agni Mitra

**Affiliations:** 1 Zoological Survey of India, New Alipore, Kolkata, West Bengal 700053 India; 2 State Forensic Science Laboratory, Junga, Shimla, Himachal Pradesh 171218 India; 3 Zoological Garden, Alipore, Kolkata, West Bengal 700027 India; 4 Wildlife Crime Control Bureau, Eastern Region, Kolkata, West Bengal 700020 India

**Keywords:** PowerPlex^®^ 21 System, Microsatellites, Cross-species amplification, Chimpanzees, Individual identification, Heterologous markers

## Abstract

**Objective:**

This study was aimed to test the PowerPlex^®^ 21 System (Promega, USA), used for human identification applications for its positive cross-species applicability in Chimpanzees (*Pan troglodytes*) in order to identify heterologous STRs which can be used for individual identification, paternity testing, relatedness establishment and reconstruction of pedigrees and studbook records for captive and wild chimpanzee breeding populations.

**Results:**

Of 21 STRs in PowerPlex^®^ 21 System (Promega, USA), 19 loci amplified and found to be polymorphic. Locus *Aml* showed differential banding patterns in males and females similar to those seen for humans and correctly assigned sexes of known identity. Altogether, 58 different alleles were found with an average 3.05 ± 0.28 alleles per locus. Mean observed (*H*o), and expected heterozygosity (*H*e) were 0.93 ± 0.03 and 0.52 ± 0.05, respectively.

## Introduction

Genetic assessment of free-ranging and captive populations is the key step towards effective formulation and prioritization of breeding policies in any conservation action plan. A few studies have provided direct evidence for an association between genetic variability and reproductive performance in natural populations [1, 2]. However, due to the limited technical expertise available with the zoo authorities, there is limited information about the genetic composition of the founders and potential breeders often limits formulation of an effective conservation plan. Studies suggest that descendants of small founders are more likely to suffer from inbreeding depression [3–5] and are more susceptible to various diseases and parasites [3, 6] Although several previous studies have reported STRs for chimpanzee [7–9], utilization of cross-species markers still provides an easy and rapid solution for profiling closely related species. Hence, data from a similar set of markers can be used in studying phylogeny, evolution and genetic divergence among closely related species [10–13]. In this study, we used PowerPlex^®^ 21 System kit (Promega, USA) designed for humans to test for cross-amplification of the 21 STRs in chimpanzees.

In this study, we identified 19 loci from the PowerPlex^®^ 21 System (Promega, USA) that cross-amplified in a single multiplex reaction.

## Main text

### Methods

#### Sample collection and DNA extraction

Hair samples from three Chimpanzees (two males and one female) were provided by the Zoo Officials in India. The samples were collected on commercially available hair sample collection cards, *GeneSeek* (http://www.neogen.com/geneseek). Genomic DNA was extracted from hair follicles using Qiagen DNeasy Blood and Tissue kit (Qiagen, Germany) following the manufacturer’s instructions. All DNA extraction procedures were done in aseptic conditions.

#### PCR and microsatellite genotyping

A commercial kit (PowerPlex^®^ 21 System, Promega, USA) designed for humans was used to amplify 21 loci in chimpanzees. The PowerPlex^®^ 21 System is used for human identification applications including forensic analysis, relationship testing and research use. The system allows co-amplification and four-color fluorescent detection of 21 loci (20 STR loci and Amelogenin), including D1S1656, D2S1338, D3S1358, D5S818, D6S1043, D7S820, D8S1179, D12S391, D13S317, D16S539, D18S51, D19S433, D21S11, Amelogenin (henceforth *Aml*), CSF1PO, FGA, Penta D, Penta E, TH01, TPOX and vWA [14–16]. PCR was conducted with the PowerPlex^®^ 21 5X Master Mix, PowerPlex^®^ 21 5X Primer Pair Mix and sterile water, amplification grade, following manufacturer’s instructions (Promega, USA). We also included positive and negative control reactions to monitor PCR contamination. All the amplification reaction were performed on GeneAmp 9700 thermocycler (Applied Biosystems, USA) in a total volume of 25 µl. Thermal cycling condition was followed as per the PowerPlex^®^ 21 System (Promega, USA). The amplified PCR products were subjected for the fragment analysis on the ABI 3130 Genetic Analyzer (Applied Biosystems, USA).

#### Statistical analysis

Raw data was processed for sizing of alleles using GeneMapper 3.7 (Applied Biosystems, USA). After scoring and re-arrangement of multi-locus genotype data was processed further for the assessment of genetic diversity estimates. Estimates of genetic diversity, including observed number of alleles per locus (Na), effective number alleles (Ne), inbreeding coefficient (F), observed (Ho) and expected heterozygosity (He), were obtained using using GENEALEX version 6.41 software [17].

### Results

Out of 21 STRs in the PowerPlex^®^ 21 System (Promega, USA), two loci (D5S818 and D12S391) failed to amplify across the chimpanzee samples and 19 loci were found to be polymorphic. Locus *Aml*, a non-STR marker, demonstrates differential banding patterns in males and females humans [18, 19], and a similar pattern was seen for chimpanzees, with females showing a 78 bp allele and the male 78 bp and 84 bp alleles. Thus, the PowerPlex^®^ 21 System has discriminating power in correctly assigning sexes in Chimpanzees, hence an extended developmental validation of the PowerPlex^®^ 21 System.

Altogether, 58 different alleles were found across 19 loci. The number of observed alleles ranged from 5 (D1S1656, D2S1338 & D19S433) to 1 (TH01), with an overall mean number of alleles per locus of 3.05 ± 0.28. The observed number of alleles for all loci exceeded the effective number of alleles, with mean observed 3.05 ± 0.28 (Table 1). Mean Observed (*H*o), and expected heterozygosity (*H*e) were 0.93 ± 0.03 and 0.52 ± 0.05, respectively. The mean unbiased expected heterozygosity (UHe) was 0.63 ± 0.06.. The fixation index (F), a representation of inbreeding was in negative, i.e., − 0.89 ± 0.17, representing an outbred population. Observed profiles of seven loci, (D1S1656, D16S539, D2S1338, D21S11, D7S820, D8S1179, and D19S433) exhibited to be relatively more heterozygous and showed numerous alleles, i.e. ≥ four alleles/locus, indicating a strong signature that these three animals are genetically unrelated and might have come from the different genetic tracts.

**Table 1:** Genetic diversity estimates of Chimpanzees with PowerPlex^®^ 21 System (Promega, USA)

Locus	Na	Ne	Ho	He	UHe	F
AMEL	2.00	1.80	1.00	0.44	0.53	− 1.25
D3S1358	3.00	2.57	1.00	0.61	0.73	− 0.64
D1S1656*	5.00	4.50	1.00	0.78	0.93	− 0.29
D6S1043^+^	4.00	3.00	1.00	0.67	0.80	− 0.50
D13S317	3.00	2.57	1.00	0.61	0.73	− 0.64
Penta E	2.00	1.38	0.66	0.28	0.33	− 1.40
D16S539^+^	4.00	3.60	1.00	0.72	0.87	− 0.39
D18S51	2.00	2.00	1.00	0.50	0.60	− 1.00
D2S1338*	5.00	4.50	1.00	0.78	0.93	− 0.29
CSF1PO	2.00	1.80	1.00	0.44	0.53	− 1.25
Penta D	2.00	1.38	1.00	0.28	0.33	− 2.60
TH01	1.00	1.00	1.00	0.00	0.00	#N/A
vWA	2.00	1.38	0.67	0.28	0.33	− 1.40
D21S11^+^	4.00	3.60	0.67	0.72	0.87	0.08
D7S820	3.00	2.57	1.00	0.61	0.73	− 0.64
TPOX	2.00	1.38	1.00	0.28	0.33	− 2.60
D8S1179	3.00	2.00	1.00	0.50	0.60	− 1.00
D19S433*	5.00	4.50	0.67	0.78	0.93	0.14
FGA^+^	4.00	3.60	1.00	0.72	0.87	− 0.39
Mean (SE)	3.05 ± 0.28	2.58 ± 0.26	0.93 ± 0.03	0.52 ± 0.05	0.63 ± 0.06	− 0.89 ± 0.17

Where, Na, the Observed number of alleles; Ne, the effective number of alleles; Ho, observed heterozygosity; He, expected heterozygosity; UHe, unbiased expected heterozygosity; F, fixation index

Loci with star (*) and plus (^+^) notion were relatively more heterozygous and can be used in discriminating genealogical relationship

Seven loci were relatively more heterozygous and contained ≥ four alleles/locus (Table 2), indicating a strong signature that these three animals are genetically unrelated and might have come from different genetic background.

**Table 2:** Genotypes of Chimps with most heterozygous STR loci

Chimp	Unique genotype ID
Male Chimp 1	72172107107248252185197297301117117194214
Female Chimp	115618091103244256197209289297103108220220
Male Chimp 2	16416899103256266193193289289117117222234

D1S1656, D16S539, D2S1338, D21S11, D7S820, D8S1179, and D19S433

### Discussion

This study has been the first attempt to establish human identification STR kit- PowerPlex^®^ 21 System (Promega, USA) in chimpanzee, extending developmental validation of the PowerPlex^®^ 21 System for individual identification and molecular sexing of chimpanzees. The results showed high genetic variability in the analyzed individuals and three chimps were genetically distinct and with no evidence of inbreeding.

This system can be used for population estimation following capture-mark-recapture methods with larger sample sizes, assigning population genetic structure in wild populations, demographic history, and investigating gene flow and other associated parameters of Chimps in wild and captivity.

## Limitations

This study identified heterologous microsatellites for Chimps and include as such no potential limitations. However, if we had more number of chimpanzee samples (reference and pellets), we could certainly propose the combination of microsatellites based on probability of identity (P_ID_) and probability of identity for siblings (P_SIB_) that can be used for individual identification through mark-recapture analysis.

## References

[1] Keller LF, Arcese P, Smith JNM, Hochachka WM, Steams SC (1994). Selection against inbred song sparrows during a natural population bottleneck. Nature.

[2] Briskie JV, Mackintosh M (2004). Hatching failure increases with severity of population bottlenecks in birds. Proc Natl Acad Sci USA.

[3] Frankham R (1995). Conservation genetics. Annu Rev Genet.

[4] Saccheri I, Kuussaari M, Kankare M (1998). Inbreeding and extinction in a butterfly metapopulation. Nature.

[5] Frankham R, Ballou JD, Briscoe DA (2002). Introduction to conservation genetics.

[6] Hale KA, Briskie JV (2007). Decreased immunocompetence in a severely bottlenecked population of an endemic New Zealand bird. Anim Conserv.

[7] Goossens B, Latour S, Vidal C, Jamart A, Ancrenaz MW, Bruford M (2000). Twenty new microsatellite loci for use with hair and faecal samples in the chimpanzee (*Pan troglodytes*). Folia Primatol.

[8] Vigilant L (2002). Technical challenges in the microsatellite genotyping of a wild chimpanzee population using feces. Evol Anthro Suppl.

[9] Becquet C, Patterson N, Stone AC, Przeworski M, Reich D (2007). Genetic structure of chimpanzee populations. PLoS Genet.

[10] Mukesh, Sathyakumar S (2011). Eighteen polymorphic microsatellites for domestic pigeon *Columba livia* var. *domestica* developed by cross species amplification of chicken markers. J Genet.

[11] Mukesh, Javed R, Gaur U, Jianlin H, Sathyakumar S (2011). Cross-species applicability of chicken microsatellite markers for investigation of genetic diversity of Indian duck *Anas platyrhynchos* populations. Afr J Biotech.

[12] Thakur M, Rai ID, Mandhan RP, Sathyakumar S (2011). A panel of polymorphic microsatellite markers in Himalayan monal *Lophophorus impejanus developed* by cross-species amplification and their applicability in other Galliformes. Eur J Wildlife Res.

[13] Mukesh, Garg S, Javed R, Sood S, Singh H (2016). Genetic evaluation of ex situ conservation breeding projects of cheer pheasant (*Catreus wallichii*) and western tragopan (*Tragopan melanocephalus*) in India. Zoo Biol.

[14] Butler JM (2006). Genetics and genomics of core STR loci used in human identity testing. J For Sci.

[15] Hill CR, Kline MC, Coble MD, Butler JM (2008). Characterization of 26 miniSTR loci for improved analysis of degraded DNA samples. J For Sci.

[16] Lu DJ, Liu QL, Zhao H (2011). Genetic data of nine non-CODIS STRs in Chinese Han population from Guangdong Province, Southern China. Int. J Legal Med..

[17] Peakall R, Smouse PE (2006). GENALEX 6: genetic analysis in Excel. Population genetic software for teaching and research. Mol Ecol Notes.

[18] Yamauchi K, Hamasaki S, Miyazaki K, Kikusui T (2000). Sex determination based on faecal DNA analysis of the amelogenin gene in Sika Deer (*Cervus nippon*). J Vet Med Sci.

[19] Pfeiffer I, Brenig B (2005). X- and Y-chromosome specific variants of the amelogenin gene allow sex determination in sheep (*Ovis aries*) and European red deer (*Cervus elaphus*). BMC Genet.

